# Antimicrobial Effect of Fluoride-Generating Nonthermal Atmospheric Plasmas on In Vitro Triple-Species Oral Biofilms

**DOI:** 10.3390/jfb17090477

**Published:** 2026-09-19

**Authors:** Anushri Warang, Linfeng Wu, Qingsong Yu, Liang Hong, Hongmin Sun

**Affiliations:** 1 Department of Internal Medicine, University of Missouri, Columbia, MO 65211, USA; anushri.warang@health.missouri.edu; 2 Department of Public Health Sciences, Texas A&M College of Dentistry, Dallas, TX 75246, USA; linfengw@tamu.edu; 3 Department of Mechanical and Aerospace Engineering, University of Missouri, Columbia, MO 65211, USA; yuq@missouri.edu

**Keywords:** *Streptococcus mutans*, *Streptococcus sanguinis*, *Candida albicans*, dental caries, oral biofilm, nitride, nitrate, hydrogen peroxide, fluoride, nonthermal plasma

## Abstract

This study investigates the feasibility of fluoride delivery via fluoride-generating nonthermal atmospheric plasma (FNTAP) and its antimicrobial effects on in vitro triple-species oral biofilms. FNTAPs were generated by adding 5 or 10 sccm (standard cubic centimeters per min) 1,1,1,2-tetrafluoroethane (TFE) to atmospheric argon (3000 sccm) nonthermal plasma (ArNTAP). Reactive plasma species were measured in plasma-treated phosphate-buffered saline (PBS). Twenty-four-hour biofilms were constructed from *Streptococcus mutans*, *Streptococcus sanguinis*, and *Candida albicans* for assessing the antimicrobial effects of FNTAPs via viability assays and colony-forming unit (CFU) assays. FNTAP treatments significantly increased the concentrations of F^−^ and NO_2_^−^ in PBS compared to ArNTAP treatments (*p* < 0.01), and decreased the concentrations of H_2_O_2_ and NO_3_^−^ in PBS (*p* < 0.001, *p* < 0.05). 5 sccm 2 min FNTAP treatment reduced biofilm viability by 94.12 ± 1.03% (*p* < 0.001), compared to a 69.32 ± 2.05% (*p* < 0.001) reduction with ArNTAP. CFU assays showed that FNTAPs were especially efficacious against *C. albicans*. 5 sccm 3 min FNTAP treatment reduced fungal log_10_ CFUs by 4.28 ± 0.36 (*p* < 0.001). Fungal CFUs were below detectable levels for the 10 sccm 2 and 3 min groups, except for two data points in each group. In conclusion, FNTAP effectively produced measurable fluoride in PBS and significantly enhanced antimicrobial efficacy against triple-species biofilms, highlighting its potential as a multifunctional tool for caries control.

## 1. Introduction

Dental caries remains one of the most prevalent chronic diseases globally, affecting billions of people, despite being largely preventable [1,2]. Cariogenic bacteria (e.g., *Streptococcus mutans*) form dental plaque biofilms on tooth surfaces, producing acids that demineralize tooth hard tissues, eventually leading to dental caries. *S. mutans* is considered the major etiological agent disrupting enamel mineral equilibrium and has consistently shown a correlation with cases of active caries [3]. The dysbiosis of supragingival plaque with overrepresentation of *S. mutans* is considered an environment conducive to the formation of dental caries [4]. *Streptococcus sanguinis* is an early colonizer of the oral cavity, contributing to the development of complex microbial communities on dental surfaces. Dental biofilms are formed by initial bacterial colonization from 0 to 18 h by aerobic bacteria such as *S. sanguinis* and other mitis group streptococci. *S. sanguinis* has been consistently recovered from individuals with high incidences of dental caries [5]. This biofilm matures from 18 h to 4 days with the addition of co-aggregator bacteria such as *S. mutans*. Obligate anaerobes invade the gum margins from 4 to 7 days, with gradual hardening of the extracellular polymeric substance (EPS) [6]. The extracellular matrix in these oral biofilms enhances the resistance of encased bacteria to both host defenses and antimicrobial agents [7]. This resilience of established dental biofilms necessitates frequent mechanical removal to manage the plaque. However, the anatomy of human dentition limits the efficacy of daily brushing at home in removing dental plaque. Even with diligent oral hygiene and professional cleanings, bacteria can rapidly recolonize and restore the biomass of biofilms within days, underscoring the difficulty in maintaining long-term biofilm control with conventional means alone [7]. Oral biofilms are linked to cardiovascular disease through direct effects of oral bacteria and inflammatory responses to oral disease [8]. *S. sanguinis* has shown the potential to impair clotting processes, further solidifying the connection between oral biofilms and systemic disease [9]. Thus, maintaining oral health and preventing dental caries are important public health concerns [10].

Candida species are the most prevalent fungal genera found in the oral cavity [11]. Specifically, *C. albicans* may transition from being a commensal to a pathogen in multi-species oral biofilms [12]. *Candida albicans* has been frequently detected in the oral cavity of children with severe early childhood caries [12]. As an opportunistic fungal organism, *Candida albicans*, in conjunction with *S. gordonii*, *S. oralis*, and *S. sanguinis*, enhances bacterial colonization and biofilm formation [13,14]. *S. mutans* interacts with *C. albicans* via cell surface glucans, while mitis group streptococci such as *S. sanguinis* bind to *C. albicans* hyphae via cell-to-cell interactions to form cariogenic cross-kingdom biofilms [15]. *C. albicans* is also known to cause denture stomatitis and is fairly resistant to routine oral hygiene cleaning regimens [16]. In addition to the above-mentioned conditions, interactions between oral bacteria and *C. albicans* are also associated with endodontic infections, periodontitis, peri-implantitis and oral cancer [17]. High levels of sucrose in the oral cavity enhance the physical interaction between *C. albicans* and *S. mutans*, increasing the microbial burden and production of extracellular matrix. In vitro cospecies biofilms with *S. mutans* and *C. albicans* are known to have a higher biomass than single-species in vitro *S. mutans* biofilms. *S. mutans* and *C. albicans* are also known to synergize the virulence of plaque biofilms in vivo [18]. Novel technologies that target *C. albicans* and its cross-kingdom interactions with bacteria are necessary to control the worldwide ubiquitous prevalence of deleterious multi-species oral biofilms.

Fluoride has been the cornerstone of caries prevention and management for decades. Fluoride releasing bioactive restorative materials have been developed for use in pediatric and elderly patients [19,20]. The primary mode of action for fluoride is the inhibition of demineralization and the enhancement of remineralization of early lesions. Tooth enamel is largely composed of hydroxyapatite, which is prone to dissolution by pH fluctuations in the oral cavity. The presence of fluoride ions leads to the formation of fluorohydroxyapatite, which is further converted to fluorapatite [21]. Fluorapatite is resistant to dissolution by pH changes. However, the presence of thick plaque can limit the contact of dental enamel with fluoride. Moreover, the direct antimicrobial effect of fluoride delivered by current commercial products is limited in terms of managing oral bacteria, and is considered secondary [22]. At typical-use concentrations (e.g., 1000–1500 ppm in toothpaste), fluoride only modestly inhibits cariogenic bacteria via a short-lived suppression of bacterial growth [22,23]. Studies have demonstrated that *S. mutans* biofilms can recover their acid production shortly after brief fluoride exposure [23]. Despite extensive use of fluoride globally, a plateau in dental caries reduction in various populations has been observed for many years. There is an urgent need for innovative strategies that prevent enamel demineralization and attenuate the pathogenicity of oral biofilms.

Nonthermal atmospheric plasma (NTAP) is a novel, non-invasive modality for targeted biofilm disruption. NTAP is an ionized gas containing a rich mixture of reactive species such as reactive oxygen and nitrogen species (ROS and RNS), free radicals, and charged particles at or near room temperature. These reactive species can penetrate biofilm structures and produce rapid antimicrobial effects without causing adverse effects on skin and soft tissues [24]. Research has demonstrated the strong antimicrobial and antibiofilm effects of NTAP on oral pathogens in planktonic form or single/dual-species biofilm models [25,26,27,28,29]. In addition, NTAP can also enhance the tooth’s resistance to decay by modifying enamel surfaces, increasing resistance to demineralization, and improving fluoride uptake [30]. Recently, our research group reported for the first time that fluoride-generating nonthermal plasma (FNTAP) had superior efficacy against in vitro dual-species oral biofilms as compared to argon NTAP or fluoride alone [31].

Nevertheless, the human oral cavity houses more than 700 species of bacteria and fungi [32]. Dental caries is a polymicrobial biofilm-induced infectious disease. However, our previous publications used single-species oral biofilms or dual-species oral biofilms in the evaluation of technologies developed for managing/treating dental caries. Multi-species oral biofilms have been reported to be more resistant to treatment [33]. Therefore, there is a need to further evaluate the potency of our novel FNTAP against more complex dental biofilms.

In the present study, triple-species biofilms were constructed from pathogens that have been found to be clinically relevant in cariogenic oral biofilms: *S. mutans*, *S. sanguinis*, and *C. albicans* [12,18,34] and were used to further evaluate the effects of FNTAP from 1,1,1,2-tetrafluoroethane (TFE). The generation of various reactive species, including NO_2_^−^, NO_3_^−^, H_2_O_2_ and fluoride, was also assessed for the first time for this novel FNTAP to provide preliminary insight into the underlying mechanisms. We hypothesized that NTAP treatment would show a significant reduction in bacterial and fungal CFUs in triple-species biofilms as compared to control, similar to the results seen in our previous study with in vitro dual-species biofilms [31]. The null hypothesis was that there is no significant difference between FNTAP treatment groups and control groups.

## 2. Materials and Methods

### 2.1. Nonthermal Atmospheric Plasma Devices and Settings

The nonthermal atmospheric plasma devices used in this study have been described in our previous publications [35]. The details on the operation of Ar plasma and FNTAP used in this study have been published [31]. Briefly, the plasma brush consists of a gas chamber with a converging nozzle at one end. In the nozzle, two tungsten needles served as cathode and the ground electrode. Argon gas at a flow rate of 3000 standard cubic centimeters per minute (sccm) (Industry Grade, Airgas, Radnor Township, PA, USA) flowed through the gas chamber and served as the carrier gas for generating NTAPs. Spellman HV power supply SL60 (Spellman, New York, NY, USA) was used as the electrical energy source in a direct current mode setting at a value of 6.8 mA to ignite NTAPs, which was blown out of the nozzle by Ar gas flow to form a brush shape NTAP plume. 1,1,1,2-tetrafluoroethane (TFE) vapor was added to the Ar NTAP at 5 sccm or 10 sccm flow rates to generate a brush shape of FNTAP plume with dimensions of ~6.0 mm in length, ~4.0 mm in width, and ~1.0 mm in thickness. Two separate mass flow controllers (MKS Instruments Inc., Andover, MA, USA) were used for controlling the flow rates of Ar gas and TFE vapor. The plasma brush tip temperature was maintained as close to the human body temperature of ~37.0 °C as possible and measured using a grounded thermocouple (TJ36-CPSS-116G-3-SMPW-M, OMEGA Engineering Inc., Norwalk, CT 06854, USA) prior to data collection. For plasma treatment, the FNTAP plume tip directly touched the biofilm surfaces.

### 2.2. Assessment of Reactive Plasma Species

One and a half milliliters of phosphate-buffered saline (PBS) were added into the well of 24-well plates and exposed to Ar NTAP or FNTAPs for 2 or 3 min. This experiment was repeated three times on the same day. This process was repeated two more times on two different days. Therefore, the total sample size was 9 (independent) samples for each plasma treatment. The resulting solutions were used for assessing reactive plasma species, including F^−^, NO_2_^−^, NO_3_^−^ and H_2_O_2_ generated by the plasmas. An aliquot of the desired volume was sampled from each prior plasma-treated PBS solution and used for the analysis of each reactive plasma species after each plasma treatment process (*n* = 9, independent) per treatment group, except for the analysis of total nitrate/nitrite and NO_3_^−^ concentrations for the Ar NTAP 2 min group which had a sample size of 8 due to a missing well during analysis. The analytical methods used in the present study were well developed and have been used in numerous studies reported in the literature [36,37,38,39,40].

A fluoride ion selective electrode (Orion™ 9609BNWP, Thermo Fisher Scientific, Waltham, MA, USA) [38] was used to measure the fluoride concentrations in the plasma-treated PBS and untreated PBS samples. The baseline fluoride level in untreated PBS was analyzed at one time with nine replicates (*n* = 9). Standard calibration solutions were prepared by sequentially diluting a 100 mM fluoride standard into 10, 5, 2, 1, 0.1, and 0.05 mM F^−^ standard calibration solutions using Millipore water. Analysis started by mixing 810 µL of sample solution or standard calibration solution with 90 µL of fluoride total ionic strength adjustment buffer III (Fisher Scientific, Waltham, MA, USA) in a 24-well plate. The fluoride electrode was dipped into the mixed solution, and the millivoltage was read after the indicator of “Ready” was displayed on the pH/mV meter screen. The millivoltage was read one or two more times by pressing reading “Measurement” button on the pH/mV meter. The fluoride concentration for each sample was determined by averaging two or three voltage readings recorded for each sample against the respective standard curve. The R^2^ values for the standard curve fitting were above 0.99. It should be noted that the millivoltage readings for untreated PBS and Ar NTAP-treated PBS samples fell outside of the calibration curve. Therefore, their calculated fluoride concentrations were based on extrapolation from the standard curve and were consequently overestimated relative to their actual F^−^ concentrations.

For the analysis of H_2_O_2_ concentrations using a commercially available colorimetric assay kit for H_2_O_2_ (OxiSelect™ Hydrogen Peroxide/Peroxidase Assay Kit, Cell Biolabs Inc., San Diego, CA, USA) [39,40], the standard H_2_O_2_ solution in the kit was used to prepare 50, 25, 12.5, 6.25, 3.125, 1.5625, 0.78125, and 0 µM H_2_O_2_ standard calibration solutions via sequential dilution using the assay buffer according to the manufacturer’s instructions. The plasma-treated PBS samples and H_2_O_2_ standard calibration solutions were processed according to the assay protocol for H_2_O_2_ specified in the kit manual. After incubation for 30 min, the absorbance at 560 nm was read using a microplate reader. In addition, the baseline level in untreated PBS was analyzed in a single process with nine independent replicates (*n* = 9) for H_2_O_2_. The R^2^ values for the standard curve fitting were above 0.99. The concentration of H_2_O_2_ for each sample was determined using its 560 nm absorbance against the respective standard curve.

For the analysis of NO_2_^−^ concentrations using a commercially available colorimetric assay kit for NO_2_^−^ (Griess Reagent Kit, Invitrogen™, Waltham, MA, USA) [36], the standard nitrite solution in the kit was used to prepare 50, 25, 12.5, 6.25, 3.125, 1.5625, 0.78125, and 0 µM NO_2_^−^ standard calibration solutions via sequential dilution using Millipore water (MilliporeSigma, Burlington, MA, USA) according to the manufacturer’s instructions. Microplate assays were performed for the plasma-treated PBS samples and NO_2_^−^ standard calibration solutions in 96-well microplates according to the assay protocol specified in the kit manual, except that the amount of each agent/sample/standard calibration solution was reduced to half of the amount specified in the kit manual to allow for processing more samples and preserving the plasma-treated samples for completion of all four analyses. After incubation for 30 min, the 96-well plates were read using a microplate reader at 548 nm. In addition, the baseline level in untreated PBS was analyzed in a single process with nine independent replicates (*n* = 9) for NO_2_^−^. The R^2^ values for the standard curve fitting were above 0.99. The concentration of NO_2_^−^ for each sample was determined using its 548 nm absorbance against the respective standard curve.

For the analysis of the total nitrate/nitrite using a commercially available colorimetric assay kit for the total nitrate/nitrite (Nitrate/Nitrite Colorimetric Assay Kit, Cayman Chemical, Ann Arbor, MI, USA) [36], the nitrate/nitrite assay buffer was diluted using 98 mL of Millipore water (MilliporeSigma, Burlington, MA, USA) to prepare 1× nitrate/nitrite assay buffer. The nitrate standard, nitrate reductase enzyme preparation and nitrate reductase cofactor preparation were dissolved using 1× assay buffer according to the kit manual. The nitrate reductase enzyme solution and nitrate reductase cofactor solution were split into four 2 mL microtubes with 300 µL per microtube. The prepared solutions were stored at low temperatures according to the kit manual. The nitrate standard solution prepared by dissolving the nitrate standard using 1× assay buffer was used to prepare 87.5, 75, 62.5, 50, 37.5, 25, 12.5, and 0 µM NO_3_^−^ standard calibration solutions via sequential dilution using 1× assay buffer according to the kit manual. Assays were performed for the plasma-treated PBS samples and NO_3_^−^ standard calibration solutions in 96-well microplates according to the assay protocol for determination of total nitrate/nitrite specified in the kit manual, except that the amount of each agent/sample/standard calibration solution was reduced to half of the amount specified in the kit manual to allow for processing more samples and preserving the plasma-treated samples for completion of all four analyses. After the final incubation for 10 min, the 96-well plates were read using a microplate reader at 548 nm. In addition, the baseline level in untreated PBS was analyzed in a single process with eight independent replicates (*n* = 8) for total nitrate/nitrite. The R^2^ values for the standard curve fitting were above 0.99. The total nitrate/nitrite concentration for each sample was determined using its 548 nm absorbance against the respective standard curve.

The NO_3_^−^ concentration in each sample was calculated by subtracting the NO_2_^−^ concentration from the total nitrate/nitrite concentration found in the same sample. Baseline levels of untreated PBS were subtracted to calculate the fluoride, H_2_O_2_, nitrite, total nitrate/nitrite and nitrate concentrations for plasma-treated PBS. It should be noted that the concentrations for fluoride (detection limit 0.02 ppm), H_2_O_2_ (detection limit 0.8 µM), NO_2_^−^ (detection limit 1.0 µM) and total nitrate/nitrite (detection limit 0.70 µM) in untreated PBS samples were below the detection limits of the corresponding methods.

### 2.3. Biofilm Culture

Single colonies of *S. mutans* (ATCC 700610, ATCC, Manassas, VA, USA) and *S. sanguinis* (ATCC 10556, ATCC, Manassas, VA, USA) were cultured separately in brain heart infusion (BHI) broth (Sigma-Aldrich, St. Louis, MO, USA) at 37 °C with 5% CO_2_ overnight. A single colony of *C. albicans* (BEI Resources, NIAID, NIH: *Candida albicans*, Strain P37005, NR-29447, Bethesda, MD, USA) was cultured in Sabouraud dextrose liquid medium (SDB) (Oxoid, Basingstoke, Hampshire, UK) at 37 °C under 5% CO_2_ overnight. Optical density at 600 nm (OD600) was measured for the resulting bacterial and fungal suspensions. Subsequently, OD600 for each suspension was separately adjusted to 0.1 using Tryptic Soy Broth (Sigma-Aldrich, St. Louis, MO, USA) supplemented with 0.6% Yeast Extract (TSYEB) (BD, East Rutherford, NJ, USA). The approximate CFUs at 0.1 OD600 were 10^8^/mL, 10^7^/mL and 10^6^/mL for *S. mutans*, *S. sanguinis* and *C. albicans,* respectively. The three resulting microbial suspensions were mixed in equal volumes to form a triple-species bacto–fungal seeding suspension, which was supplemented with 0.5% sucrose. The addition of sucrose to growth media is an established caries-related in vitro microcosm biofilm model [41,42]. The addition of 0.5% sucrose was reported to be within the range for maximum biofilm adhesion and biomass formation for *S. mutans* biofilms [43]. Wells in 24-well plates were coated with artificial saliva (1700–0305 Artificial Saliva-medical & dental research, Stabilized; Pickering Laboratories, Mountain View, CA, USA) before use as reported [31]. Briefly, 300 µL of artificial saliva was added to each well of the 24-well plate and the plate was incubated at 4 °C overnight. The artificial saliva was removed from the wells after incubation and the triple-species seeding suspension was added to each coated well at 1 mL per well. These microplates were incubated at 37 °C with 5% CO_2_ for 24 h to form in vitro triple-species biofilms. The resulting in vitro biofilms were washed twice using 500 µL of Dulbecco’s phosphate-buffered saline (DPBS) (Thermo Fisher, Waltham, MA, USA) for 3 min each on a benchtop shaker before receiving treatment.

### 2.4. Destruction of In Vitro Triple-Species Biofilms by FNTAP

The washed in vitro triple-species biofilms were assigned to 7 groups and treated according to the group assignment: negative control group receiving no plasma treatment (no-treatment group); Ar 2 min group and Ar 3 min group exposed to argon NTAP for 2 min or 3 min, respectively; TFE 5 sccm 2 min group and TFE 5 sccm 3 min group exposed to FNTAP generated by adding 5 sccm (standard cubic centimeters per min) TFE to argon for 2 min or 3 min, respectively; and TFE 10 sccm 2 min group and TFE 10 sccm 3 min group exposed to FNTAP generated by adding 10 sccm TFE to argon for 2 or 3 min, respectively. A non-plasma soluble fluoride control was excluded as fluoride is shown to possess antibiofilm activity at concentrations of 300 ppm and higher [44], which we hypothesized were unlikely to be produced by FNTAPs. Three independent experiments were conducted with three individual replicates each for the PrestoBlue™ assay and colony-forming unit assay described below *(n* = 9).

### 2.5. Prestoblue Assay

The PrestoBlue™ (Invitrogen, Waltham, MA, USA) assay was used to evaluate the metabolic activity of microorganisms within the biofilms right after treatment, as described in our previous report [31]. Briefly, 300 µL of PrestoBlue™ reagent was added to each well of the 24-well microtiter plate and the plate was incubated at 37 °C with 5% CO_2_ for 30 min as per kit instructions. After incubation, 100 µL of the reagent was removed from each well and transferred to a 96-well microtiter plate and absorbance at 600 nm and 570 nm was measured. The microbial metabolic activity of the biofilms was calculated as per kit instructions. The metabolic activity of the no-treatment group was considered 100%, and the relative metabolic activity for each of the treatment groups was calculated.

### 2.6. Colony-Forming Units (CFU) Assay

CFU assays were conducted to count the total viable bacteria (*S. mutans* and *S. sanguinis*) and the total viable fungi (*C. albicans*) for each treated biofilm after the PrestoBlue Assay [31]. The total viable bacterial count was determined using the protocol described in our previous report [31]. Briefly, the biofilms were scraped from the bottom of the 24-well plates using autoclaved wooden dowels into the 200 uL solution left behind in each well after the PrestoBlue analysis. The biomass was homogenized in the PrestoBlue reagent by pipetting up and down to create a homogeneous suspension. Each suspension was serially diluted and plated on Mitis-Salivarius Agar (MSA) (Sigma-Aldrich, St. Louis, MO, USA) plates supplemented with 1% potassium tellurite (Neogen, Lansing, MI, USA). The plates were incubated at 37 °C with 5% CO_2_ for 72 h before bacterial CFUs were counted. The total viable fungal count for each treated biofilm was determined by inoculating serial dilutions of harvested biofilms on Sabouraud Dextrose Agar (SDA) (Oxoid, Basingstoke, Hampshire, UK) plates after incubation at 37 °C with 5% CO_2_ for 24 h. Pilot experiments were conducted to ensure that MSA was selective for bacterial colonies and SDA was selective for fungal colonies.

CFU counts were log-transformed. For all dilutions, 10 µL of the biofilm suspension were spread onto agar plates from a total suspension volume of 200 µL. For bacterial CFU counts, the dilutions plated for FNTAP treatment groups were 10^−1^, 10^−2^ and 10^−3^; the dilutions plated for Ar NTAP treatment groups were 10^−2^, 10^−3^ and 10^−4^; and the dilutions plated for no-treatment groups were 10^−3^, 10^−4^ and 10^−5^. For fungal CFU counts, the dilutions plated for FNTAP treatment groups were undiluted, 10^−1^ and 10^−2^; the dilutions plated for Ar NTAP treatment groups were 10^−1^, 10^−2^ and 10^−3^; and the dilutions plated for no-treatment groups were 10^−2^, 10^−3^ and 10^−4^. CFUs were calculated by multiplying the colony count by the corresponding dilution factor and a factor of 20 to account for the ratio of the total suspension volume to the plated volume. When no colonies were observed at the lowest plated dilution, a colony count of 1 was assigned for calculation purposes. The accuracy of the finding of no growth at a given dilution was verified by corelating it with the PrestoBlue reading for the same well. To standardize the different starting baselines across experiments, the log_10_ reduction in CFU for total viable bacterial and fungal counts was calculated. The log_10_ reduction was determined by subtracting the CFU log_10_ value of each treatment from the mean CFU log_10_ value of the corresponding negative control (i.e., no-treatment) group within the same experiment.

### 2.7. Statistical Analysis

Numerical data were analyzed using GraphPad Prism 11 (GraphPad Software Inc., Boston, MA, USA) and reported as mean ± standard error. One-way ANOVA with Tukey’s post hoc test was used to analyze the statistical differences among groups. For the reactive species assay, FNTAP-treated groups were compared with the respective Ar NTAP-treated groups. For CFU assay, Ar NTAP- and FNTAP-treated groups were compared with the no-treatment groups. Spearman correlation analysis was conducted to explore the association between the mean log_10_ CFU reductions observed for each treatment and the mean levels of reactive species generated by the respective NTAPs. The statistical significance level was set at *p* = 0.05.

## 3. Results

Data for all results are available in the Supplementary Materials.

### 3.1. Generation of Reactive Species by NTAP

**Figure 1:**
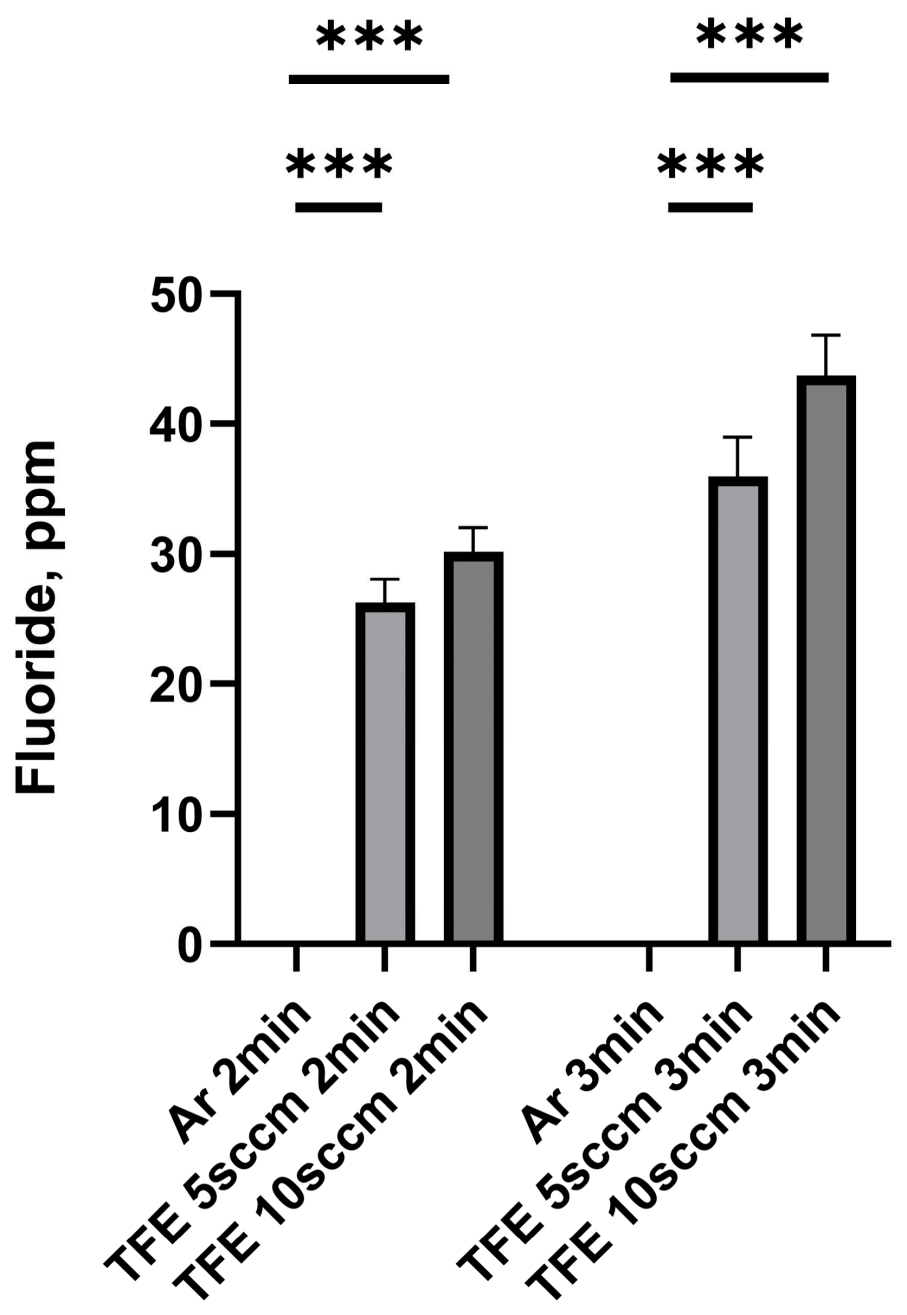
Fluoride concentrations in PBS treated with Ar NTAP or FNTAP for 2 or 3 min. Sample size: 3 independent experiments with 3 individual replicates, *n* = 9 per group. *** for *p* < 0.001 as compared with that for Ar NTAP-treated for the same period. Error bars indicate standard error of the mean. Data are available in Supplementary Materials.

Figure 1 shows the concentrations of fluoride generated in PBS exposed to NTAP.

The fluoride concentrations in PBS treated with Ar NTAP for either 2 or 3 min were extremely low, at 0.007 ± 0.0008 ppm and 0.012 ± 0.002 ppm, respectively. The fluoride concentrations in PBS treated with FNTAP for 2 or 3 min were statistically significantly higher; the TFE 5 sccm 2 min, TFE 5 sccm 3 min, TFE 10 sccm 2 min and TFE 10 sccm 3 min groups had fluoride concentrations of 26.28 ± 1.78 ppm, 35.99 ± 2.99 ppm, 30.21 ± 1.81 ppm and 43.74 ± 3.06 ppm, respectively (*p* < 0.001).

**Figure 2:**
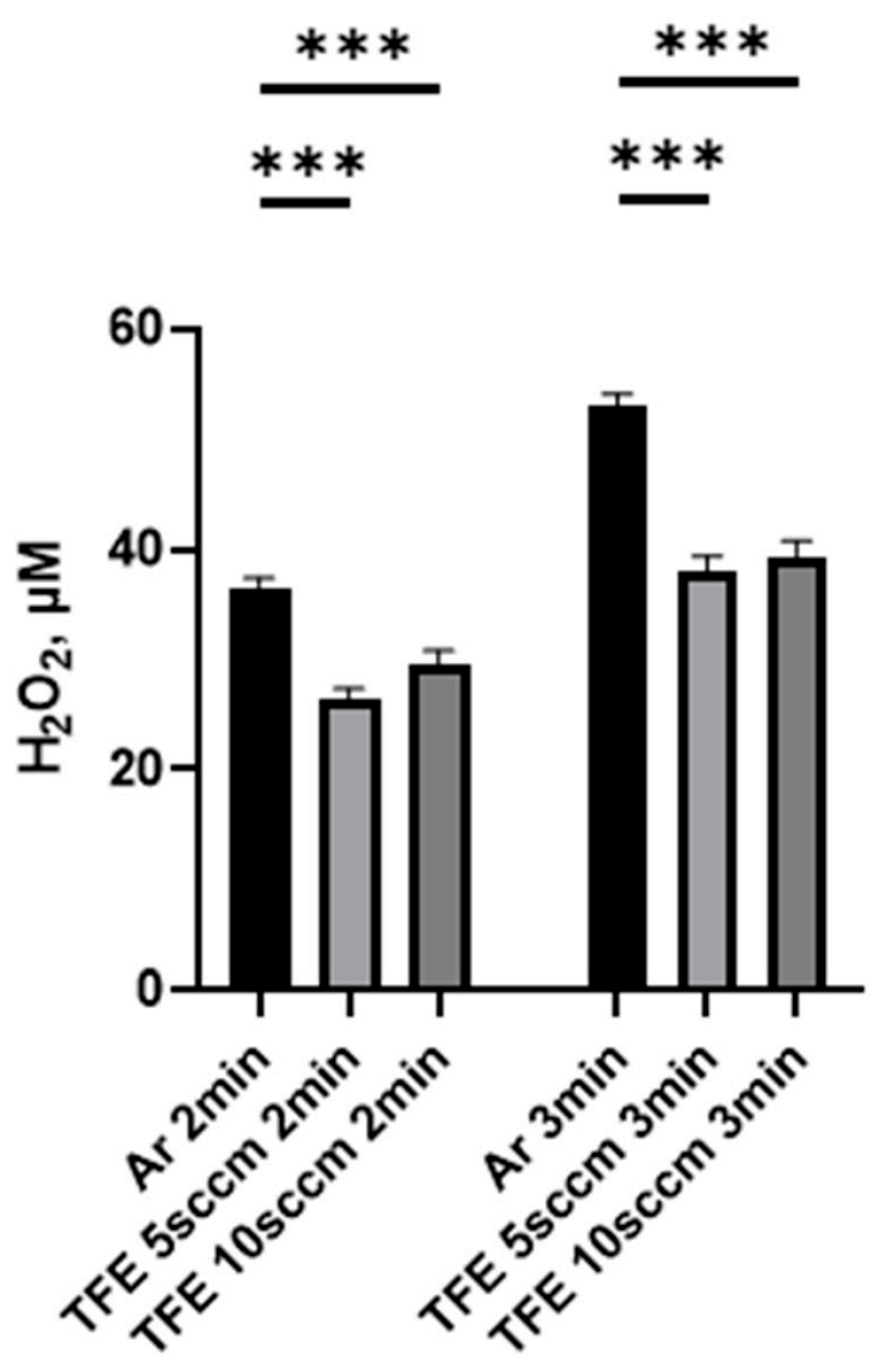
H_2_O_2_ concentrations in PBS treated with Ar NTAP or FNTAP for 2 or 3 min. Sample size: 3 independent experiments with 3 individual replicates, *n* = 9 per group. *** for *p* < 0.001 as compared with that for Ar NTAP-treated for the same time. Error bars indicate standard error of the mean. Data are available in Supplementary Materials.

The H_2_O_2_ concentration in PBS treated with Ar NTAP was 36.59 ± 0.84 µM and 53.11 ± 0.96 µM for treatment times of 2 and 3 min, respectively (Figure 2). However, the H_2_O_2_ concentrations in PBS treated with FNTAP were statistically significantly lower than those in the corresponding Ar NTAP group (*p* < 0.001) for the same exposure time; the H_2_O_2_ concentrations for the TFE 5 sccm 2 min, TFE 5 sccm 3 min, TFE 10 sccm 2 min and TFE 10 sccm 3 min groups were 26.26 ± 1.10 µM, 37.99 ± 1.38 µM, 28.32 ± 1.77 µM and 39.28 ± 1.51 µM, respectively.

**Figure 3:**
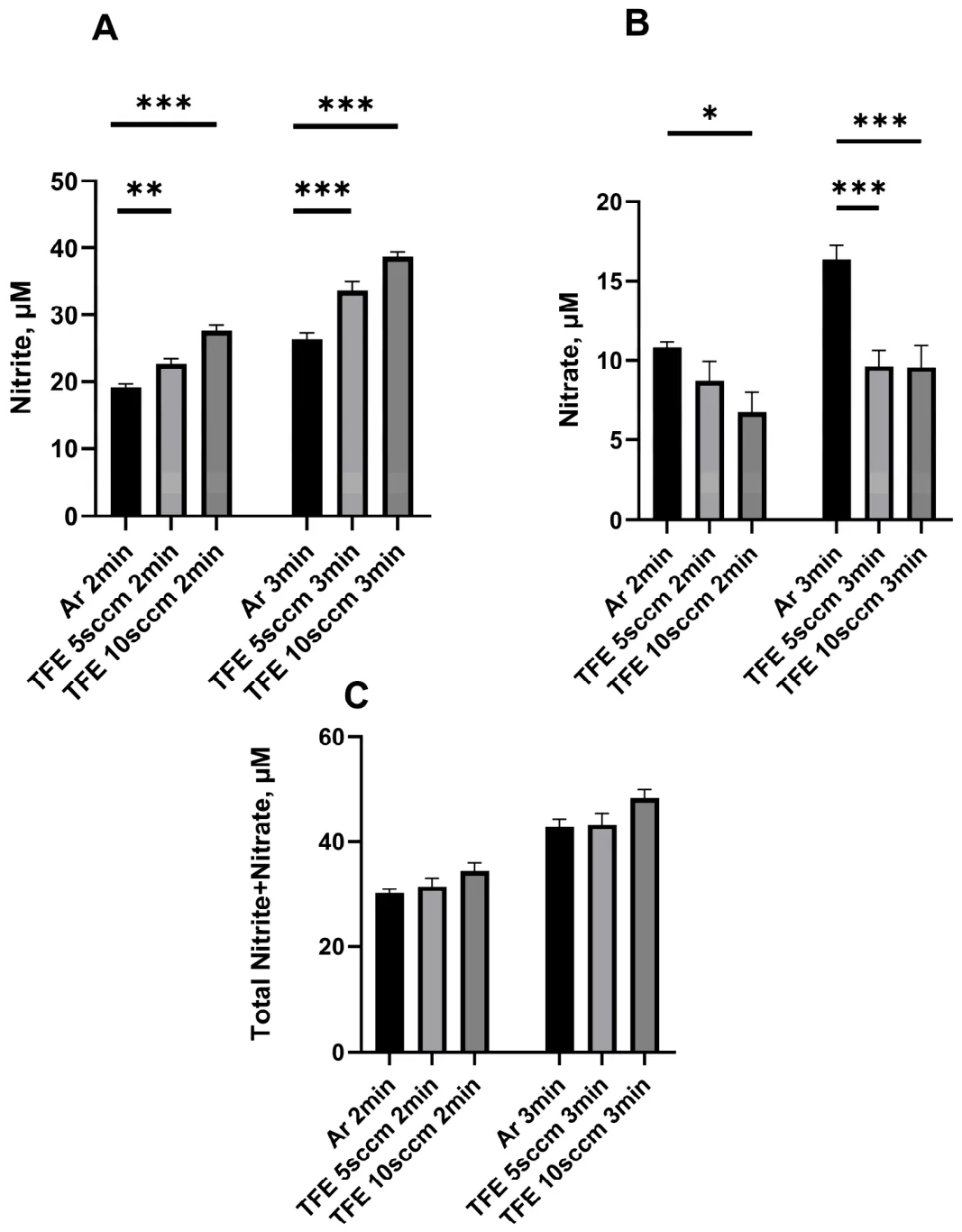
Concentrations of NO_2_^−^ (**A**), concentrations of NO_3_^−^ (**B**) and total concentrations of NO_2_^−^ and NO_3_^−^ (**C**) generated in PBS treated with Ar NTAP or FNTAP for 2 min or 3 min. Sample size: 3 independent experiments with 3 individual replicates, *n* = 9 per group, except for Ar 2 min groups for Nitrate and Total Nitrite + Nitrate analysis where *n* = 8 (3 independent experiments with 3, 3, 2 individual replicates). * for *p* < 0.05, ** for *p* <0.01, *** for *p* < 0.001 compared with that for Ar NTAP-treated for the same period. Error bars indicate standard error of the mean. Data are available in Supplementary Materials.

The NO_2_^−^ concentration in PBS was 19.19 ± 0.48 µM and 26.35 ± 0.92 µM after PBS was exposed to Ar NTAP for 2 and 3 min, respectively. The NO_2_^−^ concentrations in PBS were statistically significantly higher than those in the corresponding Ar NTAP group after PBS was exposed to FNTAP for either 2 or 3 min (*p* = 0.0049, *p* < 0.001). The nitrite concentrations for the TFE 5 sccm 2 min, TFE 5 sccm 3 min, TFE 10 sccm 2 min and TFE 10 sccm 3 min groups were 22.70 ± 0.76 µM, 33.61 ± 1.43 µM, 27.60 ± 0.91 µM and 38.75 ± 0.63 µM, respectively (Figure 3A). The NO_3_^−^ concentrations in PBS were 10.84 ± 0.33 µM and 16.37 ± 0.85 µM for the Ar 2 min and Ar 3 min groups (Figure 3B), respectively. No statistically significant difference was observed between the Ar 2 min group and the TFE 5 sccm 2 min group, which had a concentration of 8.72 ± 1.22 µM. The TFE 10 sccm 2 min group had a statistically significantly lower NO_3_^−^ concentration as compared to the Ar 2 min group: 6.73 ± 1.24 µM (*p* = 0.023). Both the TFE 5 sccm 3 min and TFE 10 sccm 3 min groups had statistically significantly lower NO_3_^−^ concentrations (9.63 ± 0.97 µM and 9.58 ± 1.39 µM, respectively) than the Ar 3 min group (*p* < 0.001, Figure 3B). Figure 3C shows the total concentrations of NO_3_^−^ and NO_2_^−^ generated in PBS exposed to NTAP. An increasing trend in the total amount of NO_3_^−^ and NO_2_^−^ generated was observed as the plasma condition changed from Ar NTAP to TFE 5 sccm FNTAP and then to TFE 10 sccm FNTAP, for both the 2 min and 3 min treatments: 30.19 ± 0.70 µM, 42.72 ± 1.54 µM, 31.42 ± 1.53 µM, 43.24 ± 2.07 µM, 34.33 ± 1.63 µM and 48.32 ± 1.54 µM, respectively, for the Ar 2 min, Ar 3 min, TFE 5 sccm 2 min, TFE 5 sccm 3 min, TFE 10 sccm 2 min and TFE 10 sccm 3 min groups.

### 3.2. Metabolic Activity of Triple-Species Biofilms Treated by NTAP

**Figure 4:**
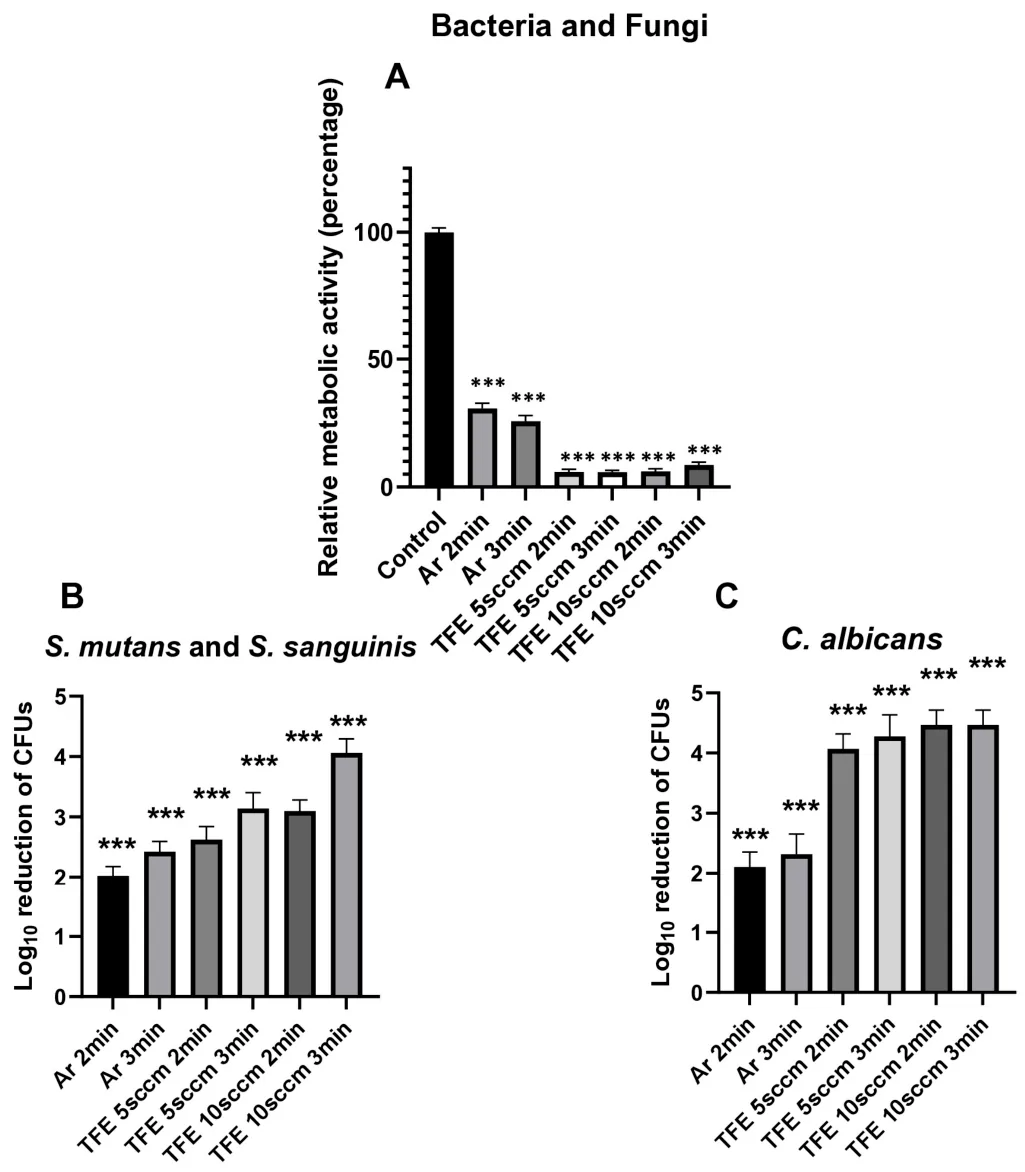
(**A**) Combined bacterial and fungal relative metabolic activity, with the metabolic activity set to be 100% for control group (i.e., untreated), *** = *p* <0.001 (all treatments compared to control). (**B**) Total bacteria CFU log10 reduction by FNTAP treatments at 2 min and 3 min treatment times, *** = *p* < 0.001 (all treatments compared to control). (**C**) Fungal CFU log10 reduction by FNTAP treatments at 2 min and 3 min treatment times,*** = *p* < 0.001 (all treatments compared to control). *n* = 9 per group (3 independent experiments with 3 individual replicates each). Error bars indicate standard error of the mean. Data are available in Supplementary Materials.

Figure 4A shows the total (bacterial and fungal) metabolic activity in in vitro triple-species biofilms after exposure to argon NTAP and FNTAPs. Ar plasma treatment resulted in a statistically significant reduction in total metabolic activity by 69.32 ± 2.05% (*p* < 0.001) and 74.32 ± 2.32% (*p* < 0.001) for the 2 and 3 min plasma exposures, respectively. FNTAP treatments reduced the total metabolic activity by 94.12 ± 1.03%, 94.22 ± 0.76%, 93.94 ± 1.05% and 91.43 ± 1.20% for the TFE 5 sccm 2 min, TFE 5 sccm 3 min, TFE 10 sccm 2 min and TFE 10 sccm 3 min, respectively (*p* < 0.001), compared to the untreated control group. Compared to Ar NTAP, the addition of TFE resulted in a statistically significantly greater reduction in total metabolic activity for both 2 and 3 min plasma exposures (*p* < 0.001). Of note, residual reactive oxygen and nitrogen species generated by nonthermal plasma may interfere with the resazurin-based PrestoBlue reaction. Therefore, CFU counts provide an independent measure that can be used to cross-validate the results of the metabolic activity assay.

### 3.3. Total Bacterial CFU Count of Triple-Species Biofilms Treated by NTAP

Figure 4B summarizes the results from total bacterial CFU assays for in vitro triple-species biofilms exposed to NTAP. Total bacterial log_10_ CFU was 6.83 ± 0.18 for the no-treatment group. Compared with the untreated control, a bacterial CFU log_10_ reduction of 2.02 ± 0.15 and 2.43 ± 0.17 was achieved following Ar NTAP treatment for 2 and 3 min, respectively (*p* < 0.001). FNTAP treatment achieved total bacterial CFU log_10_ reductions of 2.62 ± 0.22 and 3.14 ± 0.26, and 3.10 ± 0.18 and 4.06 ± 0.23 for the TFE 5 sccm 2 min, TFE 5 sccm 3 min, TFE 10 sccm 2 min and TFE 10 sccm 3 min groups, respectively (*p* < 0.001). Adding 10 sccm TFE significantly increased CFU log_10_ reductions compared to Ar NTAP treatment (*p* < 0.001) at the same treatment times.

Spearman correlation analysis was done for exploratory associations across experimental conditions and does not indicate causation. The Spearman r for the association between bacterial CFU log_10_ reduction and nitrite content in plasma-treated PBS was 0.94 (*p* = 0.0167). The Spearman r for the correlation between bacterial CFU log_10_ reduction and fluoride concentration in plasma-treated PBS was 1 (*p* = 0.0028). There was no significant correlation between bacterial CFU log_10_ reduction and the content of nitrate, total nitrates and nitrites, or H_2_O_2_ in plasma-treated PBS.

### 3.4. Fungal CFU Count of Triple-Species Biofilms Treated by NTAP

Figure 4C summarizes the results from *C. albicans* CFU assays for in vitro triple-species biofilms after plasma exposure. Total fungal log_10_ CFU was 5.77 ± 0.24 for the no-treatment group. A fungal CFU log_10_ reduction of 2.11 ± 0.25 and 2.32 ± 0.34 was achieved in the Ar 2 min and Ar 3 min groups, respectively (*p* < 0.001). With the addition of 5 sccm TFE, FNTAP treatments statistically significantly increased the fungal CFU log_10_ reduction to 4.07 ± 0.25 and 4.28 ± 0.36 after 2 and 3 min exposures, respectively (*p* < 0.001). With the addition of 10 sccm TFE, the fungal CFU counts were statistically significantly reduced, achieving a CFU log_10_ reduction of 4.47 ± 0.24 and 4.47 ± 0.24 after 2 and 3 min FNTAP treatments, respectively (*p* < 0.001). Seven out of nine CFU counts per well in each of the TFE 10 sccm 2 and 3 min FNTAP treatment groups were below the detection level (<20). Those data points were assigned the detection limit value (log_10_ 20 = 1.3) for ease of calculations.

Spearman correlation analysis was done for exploratory associations across experimental conditions and does not indicate causation. The Spearman r for the correlation between fungal CFU log10 reduction and nitrite content in plasma-treated PBS was 0.84 (*p* = 0.0444). The Spearman r for the correlation between fungal CFU log10 reduction and fluoride concentration in plasma-treated PBS was 0.90 (*p* = 0.0278). There was no significant association between fungal CFU log10 reduction and the content of nitrate, total nitrates and nitrites, or H_2_O_2_ in plasma-treated PBS.

## 4. Discussion

*C. albicans* has been frequently detected in the oral cavities of children with severe early childhood caries [12] and is routinely seen in individuals who wear dentures [34]. *C. albicans* is believed to be an important contributor to the development of early childhood caries. Studies showed that *C. albicans* enhances bacterial colonization and biofilm formation when paired with bacteria such as *S. gordonii*, *S. oralis*, and *S. sanguinis*. *C. albicans* can also synergize the virulence of plaque biofilms in vivo in connection with *S. mutans* [18]. Enhanced physical interaction between *C. albicans* and *S. mutans* due to a high level of sucrose in the oral cavity increases the microbial burden and production of extracellular matrix. Therefore, we constructed in vitro triple-species oral biofilms from *S. mutans*, *S. sanguinis*, and *C. albicans* to further assess the antimicrobial and antibiofilm potency of FNTAP. Nonthermal plasma has been shown to be effective against *C. albicans* [45]. However, the effect of FNTAP on *C. albicans*-containing oral biofilms has not been studied extensively in the literature.

Both Ar NTAP and FNTAP demonstrated strong biofilm killing potency by both metabolic activity and CFU count assays as compared to the no-treatment group. This is consistent with our previous reports on both single-species and dual-species oral biofilms [31]. For TFE 10 sccm FNTAP, fungal CFU counts were below the detectable level (<20 per well) for both groups except for two data points each. Unsurprisingly, FNTAP possessed superior antibiofilm capability against the triple-species biofilms when compared with Ar NTAP (Figure 4). This observation is consistent with our previous report on the antibiofilm potency of FNTAP on in vitro dual-species oral biofilms of *S. mutans* and *S. sanguinis* [31]. When NTAPs were used to treat dual-species biofilms for 2 min, a bacterial CFU log_10_ reduction of 2.13, >5, and >5 was observed for Ar NTAP, 5 sccm FNTAP and 10 sccm FNTAP treatments, respectively. However, for triple-species oral biofilms generated from *S. mutans*, *S. sanguinis*, and *C. albicans*, a bacterial CFU log_10_ reduction of 2.02, 2.62, and 3.10 was observed for Ar NTAP, 5 sccm FNTAP and 10 sccm FNTAP treatments, respectively, which is lower than that for dual-species biofilms. Thus, it may be inferred that *C. albicans* increased the resilience of the resulting triple-species biofilm when compared with dual-species biofilms of *S. mutans* and *S. sanguinis*. However, it should be noted that dual and triple-species biofilms were constructed in independent sets of experiments, and the comparisons should be interpreted with caution.

In this study, we achieved a fungal CFU log_10_ reduction of 2.11 ± 0.25 for *C. albicans* when the triple-species biofilms were treated using Ar NTAP for 2 min. Figueira et al. reported a log_10_ CFU/mL reduction of 1.95 for *C. albicans* in a triple-species biofilms constructed from *C. albicans*, *L. casei*, and *S.mutans* after being treated using Ar NTAP for 2 min [26]. However, adding TFE to the plasma feeding gas Ar resulted in a significant increase in the fungal CFU log_10_ reduction for *C. albicans* in triple-species biofilms treated with the 5 sccm FNTAP or 10 sccm FNTAP for either 2 or 3 min (a fungal CFU log_10_ reduction of at least 4.07 across all FNTAP treatment groups). Our findings indicate that FNTAP has strong antifungal capability, suggesting that it has potential as a topical antimicrobial treatment approach for managing fungal-associated biofilm diseases. The marked reduction in *C. albicans* in triple-species biofilms highlights the promising nature of FNTAPs for targeting bacto–fungal biofilms associated with dental caries, particularly early childhood caries and severe caries phenotypes in which *C. albicans* may interact synergistically with cariogenic bacteria such as *S. mutans*.

It is well-known that ROS and RNS contribute to the antibacterial and antibiofilm potency of NTAP when it is used to treat bacteria and biofilms [46]. In order to understand the superior antibiofilm capability of FNTAP, the reactive species generated by both Ar NTAP and FNTAP were assessed by analyzing PBS exposed to plasma. Surprisingly, lower amounts of both H_2_O_2_ and NO_3_^−^ were found in the PBS treated with FNTAPs compared with Ar NTAP, while a significantly higher amount of NO_2_^−^ was detected in PBS treated with FNTAPs. Moreover, the total RNS of NO_2_^−^ and NO_3_^−^ slightly increased for FNTAP-treated PBS. Overall, no substantial increase in ROS and RNS was observed in PBS exposed to FNTAPs. On the other hand, almost no fluoride was detected in PBS treated with Ar NTAP, while a significantly high content of fluoride was found in PBS treated by each FNTAP.

The mechanisms of FNTAP’s antibiofilm activity are currently largely unknown and need further investigation. Rather, FNTAP likely acts through a synergistic plasma-chemical mechanism. NTAP generates multiple short- and long-lived reactive species, including ROS, RNS, free radicals and charged particles, which can penetrate and disrupt the biofilm matrix, damage microbial membranes, and impair essential metabolic pathways [47]. The addition of TFE appears to shift the plasma chemistry toward increased fluoride and nitrite generation. Fluoride at this concentration may not independently kill biofilm bacteria, but in the presence of plasma-induced membrane injury, oxidative/nitrosative stress, and biofilm matrix disruption, fluoride may more readily enter bacterial cells and inhibit acid production and stress-response systems. In parallel, nitrite and other RNS may contribute to antimicrobial activity through the formation of reactive nitrogen intermediates within the chemically altered biofilm microenvironment. Therefore, FNTAP’s antibiofilm effect is best explained not as a simple fluoride-dose effect, but as a synergistic interaction among fluoride delivery, RONS-mediated biofilm disruption, microbial membrane damage. However, this is speculative, and the mechanism for this observed superior antibiofilm capability for FNTAP warrants further thorough investigation.

NTAPs have been demonstrated to promote wound healing in animal models [48,49]. While we did not evaluate the in vivo safety of FNTAPs in this study, FNTAP could potentially have broader oral and translational applications, including the topical management of denture stomatitis, oral candidiasis, and other biofilm-associated mucosal infections. Beyond the oral cavity, FNTAP could potentially be developed as a topical antifungal technology for fungal infections on other body surfaces, such as skin, nail, or mucosal infections, where localized treatment and biofilm disruption are clinically important. By generating fluorinated species together with reactive oxygen and nitrogen species, FNTAP may provide enhanced antifungal and antibiofilm activity. Therefore, FNTAP could have a promising future direction as a multifunctional topical technology for the prevention and management of fungal-associated infections after rigorous in vivo efficacy and safety studies.

We demonstrated the robust ability of FNTAP to disrupt triple-species biofilms and significantly reduce bacterial and fungal CFU counts. However, the present study has several limitations. FNTAP was tested against triple-species biofilms under in vitro conditions. Oral biofilms are substantially more complex [34]; therefore, further studies are needed to assess the efficacy of FNTAP against bacto–fungal biofilms in vivo. We selected a 24 h biofilm model for this study because it is an appropriate model for assessing oral biofilms in vitro [50] and fluoride is demonstrated to be most effective in the early stages of cariogenic oral biofilm formation [44]. This model provides an established biofilm with sufficient bacterial growth and extracellular polymeric substance production to enable evaluation of antibiofilm efficacy. In addition, the 24 h incubation period provides a practical and reproducible model for comparative evaluation of antibiofilm efficacy and is compatible with microtiter plate-based assays. Nevertheless, further studies are warranted to evaluate the antibiofilm activity of FNTAPs against more mature oral biofilms. The model used in this study is an in vitro model that cannot account for salivary flow and host hygiene practices. Plus, tooth enamel cannot be approximated by polystyrene plates. Robust in vitro cytotoxicity studies and in vivo safety studies will be necessary to evaluate the translational potential of FNTAPs. This study measured fluoride ions in FNTAP-treated PBS with limited chemical characterization of fluorinated species. Further chemical characterization of reactive plasma species is warranted in future studies. Although significant correlations were observed between reductions in bacterial and fungal CFU counts and the production of RNS and fluorine-containing species by FNTAP, these were exploratory analyses. The precise mechanisms underlying the antimicrobial activity of FNTAP remain unclear and warrant further investigation through more rigorous mechanistic studies.

## 5. Conclusions

This study demonstrates that fluoride-generating nonthermal atmospheric plasma is especially effective against *C. albicans* in in vitro triple-species biofilms. It also has the potential for significant innovation in caries prevention through its generation of fluorinated species. Beyond traditional remineralization, FNTAP could significantly enhance antimicrobial efficacy against complex oral biofilms, with its particularly superior inactivation of *C. albicans* compared with argon NTAP. These findings highlight the potential of FNTAP as a potent, multifunctional tool for caries control.

## Data Availability

The original contributions presented in the study are included in the article/Supplementary Material, further inquiries can be directed to the corresponding authors.

## Supplementary Materials

The following supporting information can be downloaded at: https://www.mdpi.com/article/10.3390/jfb17090477/s1, Table S1: Bacterial log_10_ CFU reduction as compared to no-treatment; Table S2: *C. albicans* log_10_ CFU reduction as compared to no-treatment; Table S3: Bacterial CFUs/well; Table S4: *C. albicans* CFUs/well; Table S5: Prestoblue metabolic activity analysis. Calculated as per kit instructions, no-treatment values adjusted to 1 or 100%; Table S6: Fluoride concentrations in PBS treated with Ar NTAP or FNTAP (ppm); Table S7: Concentrations of NO_2_- generated in PBS treated with Ar NTAP or FNTAP (µM); Table S8: Total concentrations of NO_2_- and NO_3_- generated in PBS treated with Ar NTAP or FNTAP (µM); Table S9: Concentrations of NO_3_- generated in PBS treated with Ar NTAP or FNTAP (µM); Table S10: H_2_O_2_ concentrations in PBS treated with Ar NTAP or FNTAP (µM).

## Abbreviations

The following abbreviations are used in this manuscript:

BHI	Brain Heart Infusion
CFU	Colony-forming units
*C. albicans*	*Candida albicans*
DPBS	Dulbecco’s Phosphate-Buffered Saline
FNTAP	Fluoride-generating nonthermal atmospheric plasma
NTAP	Nonthermal atmospheric plasma
OD600	Optical density at 600 nm
PBS	Phosphate-Buffered Saline
RNS	Reactive nitrogen species
ROS	Reactive oxygen species
sccm	Standard cubic centimeters per minute
SDA	Sabouraud Dextrose Agar
*S. sanguinis*	*Streptococcus sanguinis*
*S. mutans*	*Streptococcus mutans*
TFE	1,1,1,2-tetrafluoroethane
