# Supplementary material for: Antimicrobial Effect of Fluoride-Generating Nonthermal Atmospheric Plasmas on In Vitro Triple-Species Oral Biofilms
Source: J Funct Biomater. 2026 Sep 19;17(9):477. doi: 10.3390/jfb17090477 (PMC13608374; doi:10.3390/jfb17090477)
Supplement: Supplementary file 1 [file jfb-17-00477-s001.zip › jfb-4534468-supplementary.pdf]

Table S1

Bacterial log_{10} CFU reduction as compared to no-treatment.

	No-treatment	Ar 2min	Ar 3min	TFE 5sccm 2min	TFE 5sccm 3min	TFE 10sccm 2min	TFE 10sccm 3min
**Experiment 1**	-0.009	2.046	2.444	3.161	4.267	3.497	5.222
	0.018	2.523	3.222	3.588	4.108	3.790	4.267
	-0.009	2.523	3.222	3.653	4.046	3.775	4.523
**Experiment 2**	-0.101	1.235	2.314	2.411	3.013	2.837	3.013
	0.110	2.314	2.314	2.138	2.712	3.013	3.013
	-0.008	2.314	2.314	2.200	2.837	2.168	4.314
**Experiment 3**	-0.351	1.402	2.219	2.161	2.286	3.110	4.064
	0.064	1.950	2.110	2.219	2.763	3.161	4.064
	0.286	1.888	1.666	2.064	2.219	2.546	4.064

Table S2

C. albicans log_{10} CFU reduction as compared to no-treatment.

	No-treatment	Ar 2min	Ar 3min	TFE 5sccm 2min	TFE 5sccm 3min	TFE 10sccm 2min	TFE 10sccm 3min
**Experiment 1**	0.020	2.971	3.515	4.941	5.418	5.418	5.418
	-0.059	2.913	3.515	4.941	5.418	5.418	5.418
	0.038	3.038	3.719	4.941	5.418	5.418	5.418
**Experiment 2**	0.076	1.411	1.588	4.190	4.190	4.190	4.190
	-0.089	1.235	1.110	3.889	4.190	4.190	4.190
	0.014	1.491	1.148	3.889	4.190	4.190	4.190
**Experiment 3**	0.022	1.499	1.955	3.499	3.800	3.800	3.800
	0.022	2.499	2.198	3.499	2.076	3.800	3.800
	-0.045	1.897	2.101	2.846	3.800	3.800	3.800

Table S3

Bacterial CFUs/well.

	No-treatment	Ar 2min	Ar 3min	TFE 5sccm 2min	TFE 5sccm 3min	TFE 10sccm 2min	TFE 10sccm 3min
**Experiment 1**	34000000	300000	120000	23000	1800	10600	200
	32000000	100000	20000	8600	2600	5400	1800
	34000000	100000	20000	7400	3000	5600	1000
**Experiment 2**	5200000	240000	20000*	16000	4000	6000	4000
	3200000	20000	20000	30000	8000	4000	4000
	4200000	20000	20000*	26000	6000	28000	200
**Experiment 3**	5200000	92000	14000	16000	12000	1800	200#
	2000000	26000	18000	14000	4000	1600	200
	1200000	30000	50000	20000	14000	6600	200#

* Bacterial colonies were observed at the 10^{-2} dilution but were uncountable due to some bacterial colonies being too close together. No colonies were observed at 10^{-3} dilution; the number adjusted to 1 for calculation purposes. The dilutions plated for argon NTAP groups were 10^{-2}, $10^{-3}$and 10^{-4}.

# No bacterial colonies were observed at 10^{1} dilution. Undiluted concentration was not plated, number adjusted to 1 for calculation purposes after correlating with PrestoBlue metabolic activity analysis to ensure agreement. The dilutions plated for FTNAP groups were 10^{-1}, 10^{-2} and 10^{-3}.

For bacterial CFU counts, the dilutions plated for FNTAP treatment groups were 10^{-1}, 10^{-2} and 10^{-3}; the dilutions plated for Ar NTAP treatment groups were 10^{-2}, 10^{-3} and 10^{-4}; the dilutions plated for no-treatment groups were 10^{-3}, 10^{-4} and 10^{-5}.

Table S4

C. albicans CFUs/well

	No-treatment	Ar 2min	Ar 3min	TFE 5sccm 2min	TFE 5sccm 3min	TFE 10sccm 2min	TFE 10sccm 3min
**Experiment 1**	5000000	5600	1600	60	20*	20*	20
	6000000	6400	1600	60	20*	20*	20*
	4800000	4800	1000	60	20	20*	20*
**Experiment 2**	260000	12000	8000	20*	20*	20	20*
	380000	18000	24000	40	20*	20*	20
	300000	10000	22000	40	20*	20	20*
**Experiment 3**	120000	4000	1400	40	20*	20*	20*
	120000	400	800	40	1060	20*	20*
	140000	1600	1000	180	20*	20*	20*

* No fungal CFUs were observed at the undiluted plating concentration; the number was adjusted to 1 for calculation purposes after correlating the results with PrestoBlue metabolic activity analysis to ensure agreement.

For fungal CFU counts, the dilutions plated for FNTAP treatment groups were undiluted, 10^{-1} and 10^{-2}; the dilutions plated for Ar NTAP treatment groups were 10^{-1}, 10^{-2} and 10^{-3}; the dilutions plated for no-treatment groups were 10^{-2}, 10^{-3} and 10^{-4}.

Table S5

Prestoblue metabolic activity analysis. Calculated as per kit instructions, no-treatment values adjusted to 1 or 100%.

	No-treatment	Ar 2min	Ar 3min	TFE 5sccm 2min	TFE 5sccm 3min	TFE 10sccm 2min	TFE 10sccm 3min
**Experiment 1**	1.040	0.267	0.191	0.033	0.026	0.031	0.042
	1.050	0.236	0.180	0.031	0.035	0.034	0.054
	0.910	0.222	0.158	0.028	0.048	0.029	0.049
**Experiment 2**	1.047	0.399	0.338	0.053	0.046	0.060	0.069
	1.012	0.336	0.306	0.057	0.057	0.040	0.110
	0.941	0.349	0.354	0.053	0.057	0.059	0.074
**Experiment 3**	1.002	0.372	0.257	0.059	0.093	0.094	0.110
	1.039	0.278	0.273	0.093	0.071	0.081	0.116
	0.959	0.302	0.255	0.122	0.088	0.119	0.146

Table S6

Fluoride concentrations in PBS treated with Ar NTAP or FNTAP (ppm).

	Ar 2min	Ar 3min	TFE 5sccm 2min	TFE 5sccm 3min	TFE 10sccm 2min	TFE 10sccm 3min
**Experiment 1**	0.011	0.026	33.051	46.013	37.499	58.715
	0.010	0.020	32.623	47.026	36.532	54.765
	0.010	0.019	31.369	48.692	33.051	52.432
**Experiment 2**	0.007	0.008	27.460	34.548	27.686	39.880
	0.006	0.007	26.903	32.222	24.988	39.231
	0.005	0.007	23.498	33.847	25.194	37.348
**Experiment 3**	0.004	0.007	22.321	28.742	35.822	34.956
	0.006	0.009	20.573	26.817	26.276	33.834
	0.007	0.008	18.731	25.957	24.818	42.512

Table S7

Concentrations of NO_2^- generated in PBS treated with Ar NTAP or FNTAP (μM).

	Ar 2min	Ar 3min	TFE 5sccm 2min	TFE 5sccm 3min	TFE 10sccm 2min	TFE 10sccm 3min
Experiment 1	18.646	26.522	22.194	32.907	26.498	37.212
	18.504	23.920	23.093	30.471	25.481	36.289
	18.717	24.653	22.312	31.748	32.624	36.124
Experiment 2	21.859	30.606	26.900	36.084	28.028	41.585
	20.938	29.938	21.053	38.501	29.892	40.112
	20.155	28.948	25.703	41.585	29.984	39.606
Experiment 3	18.192	23.898	20.699	32.778	24.709	38.627
	17.714	24.900	22.250	29.078	26.094	38.842
	18.001	23.778	20.054	29.364	25.091	40.322

Table S8

Total concentrations of NO_2^- and NO_3^- generated in PBS treated with Ar NTAP or FNTAP (μM).

	Ar 2min	Ar 3min	TFE 5sccm 2min	TFE 5sccm 3min	TFE 10sccm 2min	TFE 10sccm 3min
Experiment 1	29.032	44.902	31.938	45.405	37.610	48.730
	30.625	43.505	35.822	41.968	35.570	49.037
	29.619	39.035	32.972	42.890	41.521	45.377
Experiment 2	32.169	49.491	34.846	47.742	37.496	53.070
	32.469	46.103	35.365	49.054	38.835	55.365
	32.087	47.387	35.174	53.261	30.584	51.540
Experiment 3	28.297	38.868	26.072	37.305	28.297	40.881
	27.185	39.212	26.310	35.291	29.463	43.716
		35.980	24.270	36.245	29.622	47.187

Table S9

Concentrations of NO3- generated in PBS treated with Ar NTAP or FNTAP (μM).

	Ar 2min	Ar 3min	TFE 5sccm 2min	TFE 5sccm 3min	TFE 10sccm 2min	TFE 10sccm 3min
**Experiment 1**	10.386	18.380	9.744	12.498	11.112	11.518
	12.120	19.585	12.729	11.497	10.089	12.748
	10.902	14.381	10.660	11.142	8.898	9.253
**Experiment 2**	10.310	18.885	7.946	11.658	9.469	11.485
	11.531	16.165	14.312	10.553	8.943	15.253
	11.931	18.439	9.471	11.676	0.600	11.934
**Experiment 3**	10.105	14.970	5.373	4.527	3.588	2.254
	9.470	14.312	4.060	6.213	3.369	4.874
		12.202	4.216	6.881	4.531	6.865

Table S10

H_2O_2 concentrations in PBS treated with Ar NTAP or FNTAP (μM).

	Ar 2min	Ar 3min	TFE 5sccm 2min	TFE 5sccm 3min	TFE 10sccm 2min	TFE 10sccm 3min
**Experiment 1**	33.438	53.027	28.949	39.607	33.126	44.349
	34.482	52.523	29.909	39.763	31.697	45.921
	36.379	47.949	29.801	43.028	37.099	43.136
**Experiment 2**	40.301	56.757	26.881	39.609	27.184	39.014
	40.252	55.458	28.083	38.771	28.933	38.710
	38.479	55.191	26.249	42.609	29.395	38.746
**Experiment 3**	35.854	55.328	21.587	32.981	19.925	31.917
	35.695	51.600	22.480	31.795	23.996	35.793
	34.399	50.145	22.406	33.751	23.543	35.903
